# Simplified technique in total pancreatectomy with islet cell autotransplantation after Frey’s procedure: a case report

**DOI:** 10.1186/s40792-024-02066-7

**Published:** 2024-11-27

**Authors:** Ryo Oikawa, Nobuyuki Takemura, Masayuki Shimoda, Mai Nakamura, Fuminori Mihara, Fuyuki Inagaki, Norihiro Kokudo

**Affiliations:** Hepato-Biliary-Pancreatic Surgery Division, Department of Surgery, National Center for Global Health and Medicine, 1-21-1 Toyama, Shinjuku-Ku, Tokyo 162-8655 Japan

**Keywords:** Frey’s procedure, Total pancreatectomy, Islet cell autotransplantation

## Abstract

**Background:**

The selection of the surgical approach for chronic pancreatitis (CP) is determined by various factors including inflammatory location, presence of pancreatic duct dilatation, or possibility of cancer. Total pancreatectomy (TP), with or without islet cell autotransplantation, is considered for patients with refractory CP after the failure of other surgical treatments. Considering the increasing incidence of CP requiring surgical treatment, the number of cases in which TP is performed after previous surgical treatment is expected to increase.

**Case presentation:**

We reported a case of TPIAT in a patient with alcoholic CP who had previously undergone Frey’s procedure. During the operation, the sufficient length of the elevated jejunal loop for pancreaticojejunostomy in Frey’s procedure allowed it to be used directly for biliary jejunostomy during TP. In addition, jejunojejunostomy from the previous operation could be used, and these methods contributed to simplifying the operative procedure. We need open hemostasis on post-operative day (POD) 1and a percutaneous drainage tube replacement for an intraperitoneal abscess on POD 24. The patient was discharged from the hospital on POD 37 with normal serum C-peptide level, which suggests favorable insulin secretion from transplanted islets, and the epigastric pain that suffered her preoperatively resulted in a dramatic improvement.

**Conclusions:**

When performing the Frey’s procedure, the elevated jejunal loop and Y-anastomosis jejunal loop with sufficient length allow them to be used directly for the reconstructions in the possible subsequent TP.

## Background

Chronic pancreatitis (CP) is a progressive inflammatory disease characterized by irreversible changes in the pancreatic tissue and duct obstruction, resulting in raised intraductal pressure, pancreatic ischemia, and neuroimmune interactions, leading to intractable pain, malabsorption, and diabetes [1–3]. Alcohol excess is the main cause, while other factors include biliary disease, hyperlipidemia, hyperparathyroidism, and autoimmune and familial pancreatitis [4, 5]. Treatment for CP includes behavioral modifications for risk mitigation, management of sequelae, optimal pain control involving a pain control team, and endoscopic interventions. In cases where symptoms remain refractory to these treatments, operative interventions may be considered.

Traditional operative procedures for CP include drainage procedures (e.g., Puestow procedure), resection procedures (e.g., pancreaticoduodenectomy, distal pancreatectomy, Burger procedure, and total pancreatectomy), and a combination of both procedures (e.g., Frey’s procedure) [6, 7]. Although several factors, including inflammatory location, presence of pancreatic duct dilatation, or possibility of cancer, can affect the selection of the surgical approach [6, 8], total pancreatectomy (TP), with or without islet cell autotransplantation [9], is appropriate for patients with abdominal pain recurrence after failure of surgical treatment [10]. Considering the increasing incidence of CP each year, wherein 50% of the patients with CP require surgical treatment [6], the number of cases in which TP, with or without islet cell autotransplantation, is performed after previous surgical treatment is expected to increase.

In the present report, we describe the unique surgical techniques used during total pancreatectomy with islet cell autotransplantation (TPIAT) in a patient with alcoholic CP who had previously undergone Frey’s procedure. We also discuss patients’ insulin dependence after TPIAT.

## Case presentation

**Fig. 1:**
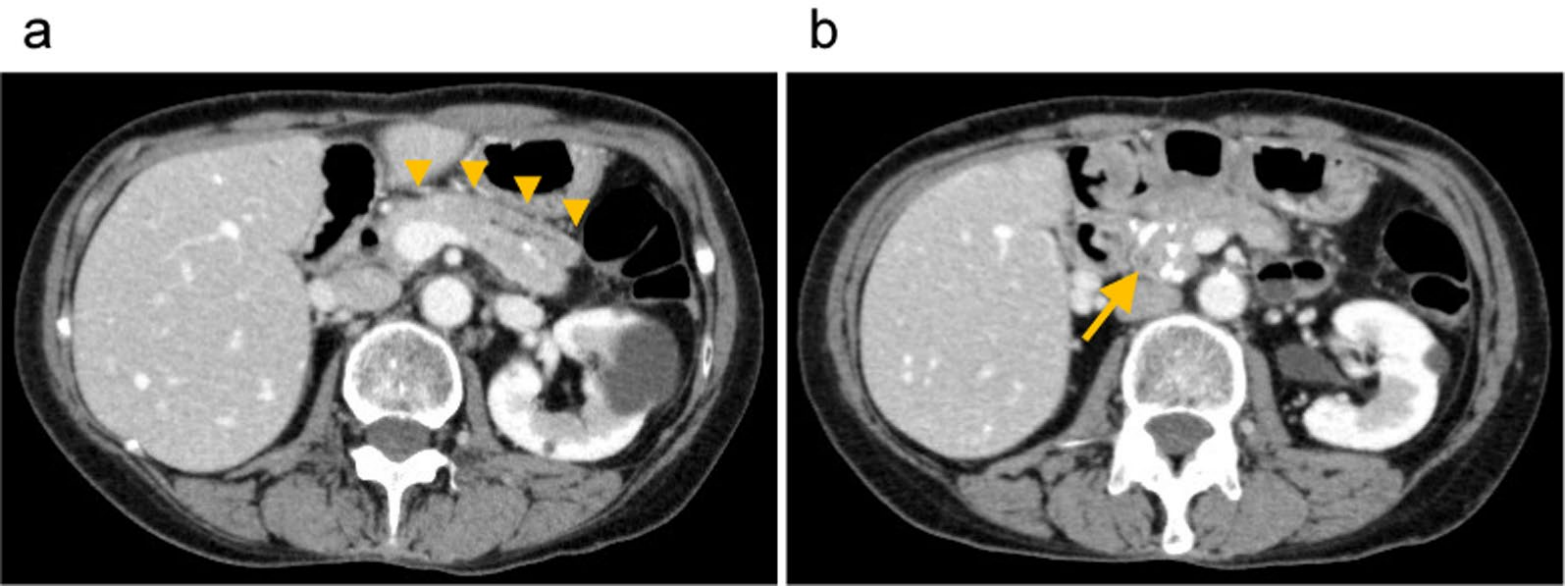
Preoperative abdominal computed tomography imagings. **a** Preoperative abdominal computed tomography imaging showing longitudinal pancreaticojejunostomy performed in the previous Frey’s procedure (arrowheads). **b** Preoperative abdominal computed tomography imaging showing multiple pancreatic calculus in the pancreatic head (arrow)

A 59-year-old woman repeatedly visited the hospital and was discharged due to epigastric pain from alcoholic chronic pancreatitis. To relieve her symptom, cyst-gastric drainage for a giant pseudocyst in the pancreatic tail and ERBD tube implantation for bile duct stenosis were performed in 2014. However, these did not provide symptom relief. The patient underwent Frey’s procedure and biliary-duodenal anastomosis to achieve symptom relief in 2017. Following a temporal reduction in her symptoms after the operation, the symptoms flared again in 2021. An ERPD tube was placed and opioids were introduced; however, they were ineffective, and the patient was admitted and discharged from the hospital again. She was referred to our hospital in 2022 and planned to undergo TPIAT to reduce the symptoms and preserve pancreatic endocrine function. Preoperative abdominal computed tomography revealed multiple pancreatic calculus in the pancreatic head (Fig. 1).

**Fig. 2:**
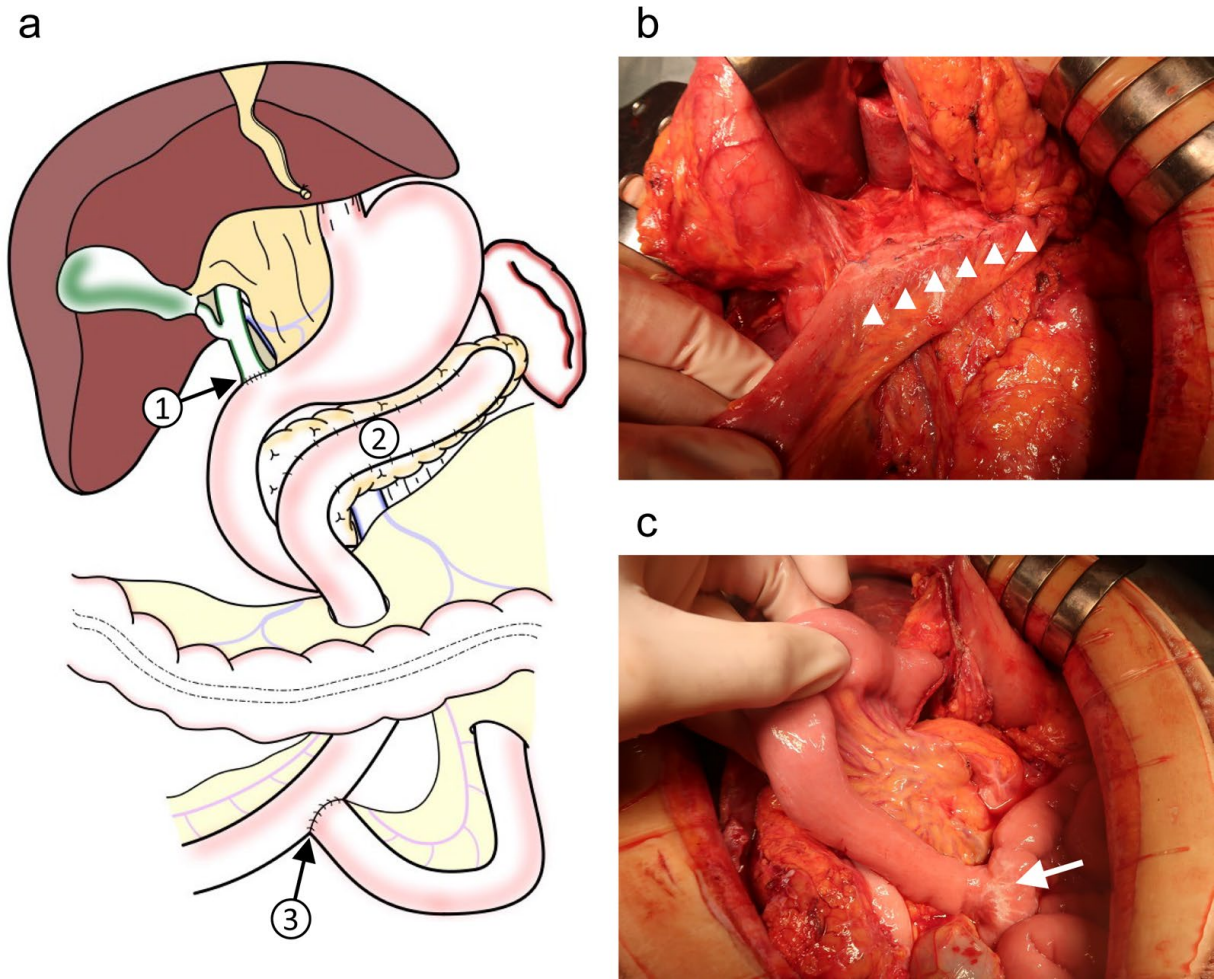
Procedures performed in the previous Frey’s procedure. **a** A schema of the previous Frey’s procedure including the biliary-duodenal anastomosis (1), longitudinal pancreaticojejunostomy (2) and jejunojejunostomy (3). **b** An intraoperative photograph of the longitudinal pancreaticojejunostomy (arrowheads). **c** An intraoperative photograph of the jejunojejunostomy (arrow)

**Fig. 3:**
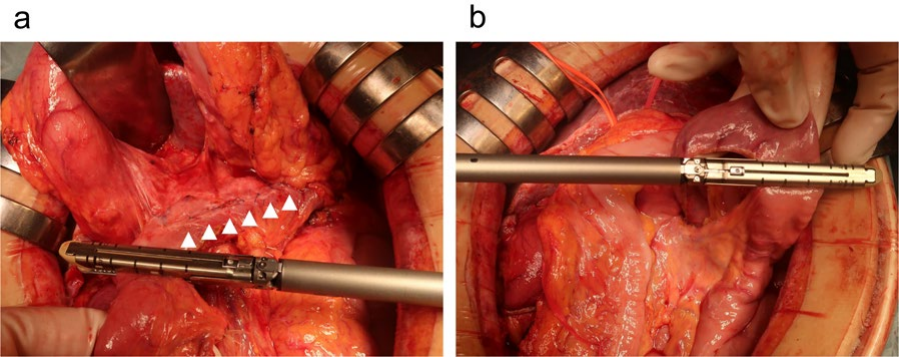
Intraoperative photographs in the present surgery. **a** An intraoperative photograph of the division of the elevated jejunal loop near the longitudinal pancreaticojejunostomy (arrowheads). **b** An intraoperative photograph of the division of the jejunum at 7 cm from the Treitz’s ligament

**Fig. 4:**
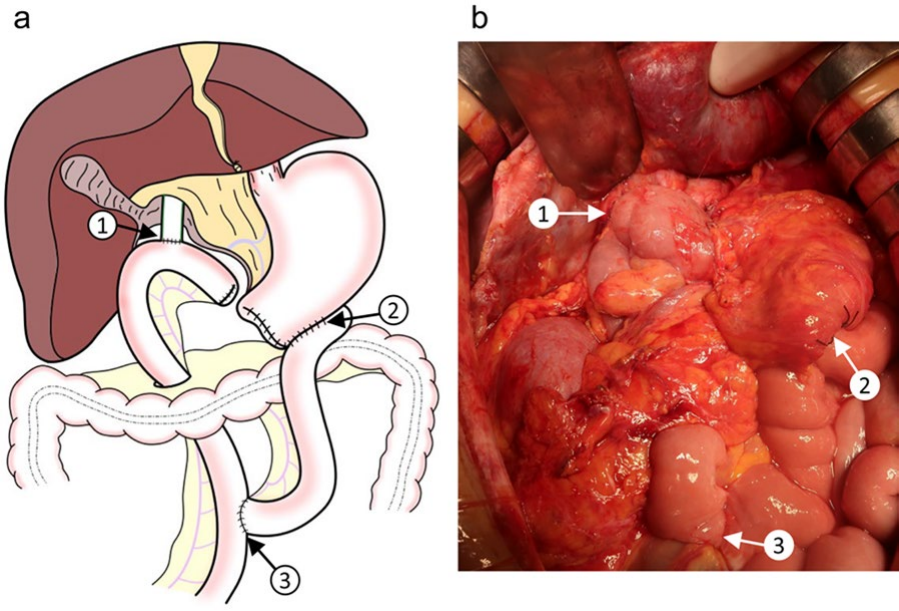
Reconstruction performed in the present surgery. **a** A schema of the reconstruction including the biliary jejunostomy (1), anterior colic gastrojejunostomy (2), and jejunojejunostomy (3). **b** An intraoperative photograph of the biliary jejunostomy (1), anterior colic gastrojejunostomy (2), and jejunojejunostomy (3)

**Fig. 5:**
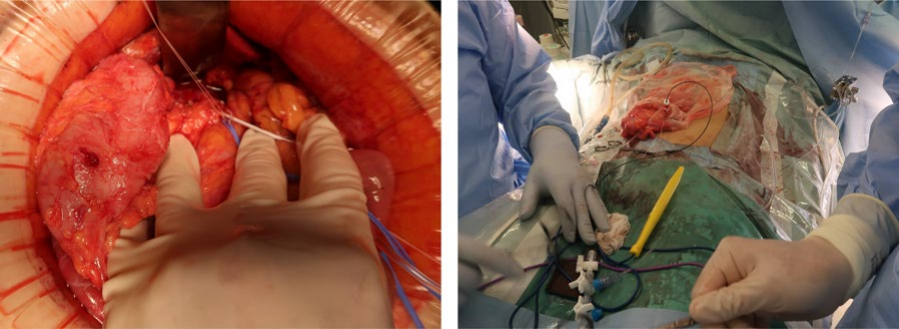
Intraoperative images of the injection route of islet cells. Extracted islet cells were injected through the sheath inserted into the main trunk of the portal vein

Longitudinal pancreaticojejunostomy and jejunojejunostomy were performed following coring out resection of the pancreatic head as part of Frey’s procedure during a previous surgery. A biliary-duodenal anastomosis was established in the distal part of the common bile duct (Fig. 2). First, the spleen and pancreatic tail were mobilized. The stomach was then divided at the proximal pylorus, and the elevated jejunal loop for the pancreaticojejunostomy was deemed possible to use for biliary jejunostomy. Therefore, it was divided near the pancreaticojejunostomy to keep it as long as possible (Fig. 3a). The common bile duct was divided at the proximal side of the biliary-duodenal anastomosis, and the jejunum was divided 7 cm from the ligament of Treitz (Fig. 3b). The pancreas and spleen were fixed with only the gastroduodenal artery, splenic artery, splenic vein, and pancreatic head plexus. After confirming that the back table was ready, the structures were ligated, and the pancreas and spleen were removed. We performed a biliary jejunostomy using the same elevated jejunal loop from the pancreaticojejunostomy in the previous operation which remained at a length of about 20 cm, and an anterior colic gastrojejunostomy using the divided jejunum near the ligament of Treitz (Y-anastomosis jejunum loop) which remained at a length of about 15 cm, with preservation of the jejunojejunostomy in the previous operation (Fig. 4). Since the patient had undergone ileocolic resection, we inserted a sheath into the main trunk of the portal vein through a branch of the middle colic vein as an injection route of islet cells (Fig. 5). After extraction, the islet cells were injected into the main trunk of the portal vein for an hour, with a total of 115,878 islet equivalent (IEQ).

Postoperatively, bloody ascites was continuously flowing through the drainage tubes, which may be due to the prolonged effect of heparin in the preservation solution of islet cells. We administered fresh frozen plasma, but progressive anemia was still observed; therefore, open hemostasis was performed on post-operative day (POD) 1. During surgery, we identified slight oozing at multiple sites and performed hemostasis. After reoperation, the patient’s condition stabilized, and the intubation tube was removed on POD 5. She was discharged from the intensive care unit on POD 10. The patient developed an intraperitoneal abscess, which was treated with a percutaneous drainage tube replacement on POD 24. After receiving education regarding diabetes and instructions on insulin administration technique, the patient was discharged from the hospital on POD 37. At the time of discharge from the hospital, she consumed three units of long-acting insulin analogue; however, laboratory data showed normal C-peptide level (0.81 ng/mL [reference range: 0.80–2.50 U/L]), which suggests favorable insulin secretion from transplanted islets, and her epigastric pain had been relieved dramatically.

## Discussion

Frey’s procedure was first introduced by Frey and Smith in 1987 as a duodenum-preserving procedure that cores out of the pancreatic head, combined with longitudinal pancreaticojejunostomy [11]. In this procedure, the complex branch ducts in the pancreatic head are decompressed by resection of the parenchyma, and the combined longitudinal pancreaticojejunostomy effectively decompresses the lateral main pancreatic duct, even in patients with multiple duct stenoses and duct dilatation. Frey’s procedure provides reasonable pain control at long-term follow-up and is associated with a better quality of life, shorter operation time, and lower rate of post-operative complications than simple pancreaticojejunostomy or classical pancreatectomy for CP [12–16]. On the other hand, 10 to 20% of patients demonstrate persistent pain after the Frey’s procedure [13]. In the present case, this procedure was performed in a patient with refractory CP who did not respond to endoscopic treatment. Although temporary relief of symptoms was achieved, the symptoms relapsed four years after the surgery.

TPIAT was first described in 1979 [17]. The major difference of this procedure from the normal total pancreatectomy is the need for a technique to minimize the warm ischemic time which is highly associated with islet survival. Ligation of blood vessels that are significantly responsible for pancreatic blood flow is delayed until the final steps of the procedure. The extracted pancreas is immediately placed in cold balanced electrolyte solution, and exsanguination of the pancreas is allowed by flushing the arterial vessels and opening the venous outflow [18]. A method to exsanguinate the pancreas by injecting cold storage fluid through the main pancreatic duct has also been reported [19]. The patient’s islet cells, isolated from the resected pancreas, are injected into the portal vein as an autologous transplant [20]. As CP patients have a high prevalence of gastroparesis, Roux-en-Y biliary jejunostomy and gastrojejunostomy is preferred in TPIAT to avoid bile reflux and afferent limb problems [18, 20]. More than 1200 TPIAT cases have been performed by 2020, mainly in Europe and the United States, according to the recent report from the Collaborative Islet Transplantation Registry (CITR) (https://citregistry.org/system/files/02nd_CITR_Network_Report_Autograft_2022.pdf), however, few cases have been performed in Asia, especially in Japan, where only one summarized report from our institution has been published [21]. Previous studies have reported the effectiveness of TPIAT, as it can improve quality of life and reduce the need for opioids in patients with CP [5, 22], while preserving the patient’s own pancreatic endocrine function. Comparing TP alone, TPIAT resulted in a significant reduction in the 24-h insulin requirement [23], with 5- and 10-year insulin independence rates of 46%–64% [24, 25] and 28% [25], respectively. Prior pancreatic surgery, impaired glycemic control before surgery, and longer duration of disease have been identified as risk factors for a low islet mass and persistent insulin dependence after TPIAT [26–29]. In the present case, although the symptoms due to CP improved well and the use of opioids was no longer necessary after surgery, the islet graft was slightly lower (3690 IEQ/kg) than the average amount reported previously [7, 18, 21], and the patient did not achieve complete insulin independence after surgery. This may be due to the long history of CP and previous treatments, including endoscopic therapy and pancreatic surgery; earlier intervention may have been better. However, if the symptoms were relieved only with Frey’s procedure, TP would not be necessary, and the timing of additional surgery would be difficult to determine. Since the patient’s C-peptide remained in the normal range requiring less insulin use after the surgery, the timing of TPIAT was not too late for the patient to benefit sufficiently from the procedure.

TPIAT after Frey’s procedure is uncommon, with CITR reporting it at about 1.5% of all TPIAT cases (https://citregistry.org/system/files/02nd_CITR_Network_Report_Autograft_2022.pdf). In fact, this may be the first report describing the method of TPIAT after Frey’s procedure in detail. However, with the increasing number of worldwide institutions performing TPIAT for patients with CP [20, 21, 30], it is expected that TPIAT after pancreatic surgeries will increasingly be performed. In the present case, 20 cm of the remaining elevated jejunal loop before the reconstruction allowed it to be used directly for biliary jejunostomy during TP. In addition, 15 cm of the remaining Y-anastomosis jejunum loop allowed the preservation of the jejunojejunostomy from the previous operation. These methods contributed to simplifying the operative procedure. Considering the small size of the patient in the present case, when performing the Frey’s procedure, it is advisable to ensure the length of the elevated jejunal loop and Y-anastomosis jejunal loop to be 20–30 cm each for possible subsequent TP.

## Conclusion

In conclusion, we describe a case of TPIAT in a patient with alcoholic CP who had previously undergone Frey’s procedure. When performing the Frey’s procedure, the elevated jejunal loop and Y-anastomosis jejunal loop with sufficient length allow them to be used directly for the reconstructions in the possible subsequent TP.

## Data Availability

The datasets analyzed during the current study are not publicly available due to their containing information that could compromise the privacy of research participants but are available from the corresponding author on reasonable request.

## Abbreviations

CITR: Collaborative islet transplantation registry
CP: Chronic pancreatitis
IEQ: Islet equivalent
POD: Post-operative day
TP: Total pancreatectomy
TPIAT: Total pancreatectomy with islet cell autotransplantation
